# Mental Health and Physical Activity in Health-Related University Students during the COVID-19 Pandemic

**DOI:** 10.3390/healthcare9070801

**Published:** 2021-06-25

**Authors:** Jasminka Talapko, Ivan Perić, Patricia Vulić, Emina Pustijanac, Melita Jukić, Sanja Bekić, Tomislav Meštrović, Ivana Škrlec

**Affiliations:** 1 Faculty of Dental Medicine and Health, Josip Juraj Strossmayer University of Osijek, HR-31000 Osijek, Croatia; jtalapko@fdmz.hr (J.T.); iperic@fdmz.hr (I.P.); pvulic@fdmz.hr (P.V.); 2 Faculty of Natural Sciences, Juraj Dobrila University of Pula, HR-52100 Pula, Croatia; emina.pustijanac@unipu.hr; 3 Faculty of Medicine, Josip Juraj Strossmayer University of Osijek, Josipa Huttlera 4, HR-31000 Osijek, Croatia; mjuki17@gmail.com (M.J.); sbekic@mefos.hr (S.B.); 4 General Hospital Vukovar, Županijska 35, HR-32000 Vukovar, Croatia; 5 Family Medicine Practice, HR-31000 Osijek, Croatia; 6 University Centre Varaždin, University North, HR-42000 Varaždin, Croatia; tmestrovic@unin.hr

**Keywords:** anxiety, COVID-19, depression, physical activity, stress, students

## Abstract

The coronavirus disease 2019 (COVID-19) pandemic led to increased negative emotional states among students. Physical activity is known to have positive impacts on mental health and well-being. However, due to the closure of gyms and other recreational facilities as a restrictive measure, students’ physical activity levels may decrease. This cross-sectional study aimed to determine the prevalence of depression, anxiety, and stress symptoms and physical activity among health-related students during the second partial COVID-19 lockdown. The study included 823 students from the Faculty of Dental Medicine and Health of the University of Osijek in Croatia. The Depression Anxiety Stress Scale-21 (DASS-21) and the Godin-Shephard Leisure Time Questionnaire for Physical Activity (GSLTPAQ) questionnaires were used to assess the prevalence of depression, anxiety, and stress symptoms as well as physical activity. Two-thirds (59.2%) of students in health-related fields were insufficiently active, while the prevalence of depression (50.8%), anxiety (50.9%), and stress (49.9%) symptoms were high. Also, female respondents had significantly higher levels of depression, anxiety, and stress than their male counterparts. Graduate students had higher levels of all three negative emotional states, but only anxiety levels were significant. This study shows that students in health-related fields had reduced physical activity and a high prevalence of negative emotional conditions (depression, anxiety, and stress) during the second partial lockdown. The resulting symptoms were mostly of mild intensity; however, we consider this a significant mental health issue during the COVID-19 pandemic. Hence, it is crucial to control and support students’ mental health, especially in more affected female individuals, in order to reduce the pandemic’s negative impact.

## 1. Introduction

The coronavirus disease 2019 (COVID-19) pandemic, caused by the severe acute respiratory syndrome coronavirus 2 (SARS-CoV-2), is a major global health issue nowadays. Numerous introduced restriction measures have affected the mental and physical health of entire populations. Given the whole situation, it is essential to maintain a certain level of physical activity. The whole-body movement or movement of individual parts of the body in a space that is enabled by skeletal muscles’ activity and whose action results in increased energy expenditure can be defined as physical activity (PA) [1]. It is well known that the adequate presence of any appropriate type of PA (e.g., walking, jogging, cycling, swimming) has a positive physiological effect and reduces the risk of non-communicable diseases, such as cardiorespiratory disorders, type 2 diabetes, hypertension, as well as other chronic diseases and comorbidities that can result in death [2,3]. Appropriate physical exercise ensures the preservation of the immune system and the general improvement of health status, mental health, and quality of life [3,4,5]. Strengthening the immune system through appropriate physical exercise can potentially reduce the spread of communicable viral infectious diseases [6]. The general trend of reduced PA levels in everyday life, sedentary lifestyle, the use of modern technology, and common adverse effects of this lifestyle on global health forced the World Health Organization to issue recommendations on physical activity for most population groups [7].

Due to the COVID-19 pandemic, restrictive measures relating to maintaining social distance between people and the complete or partial closure of economic activities are being enacted. Such a situation has contributed to the trend of physical activity deficit in the population worldwide [8,9,10]. Students are also one of the groups in society whose mental and physical condition is negatively affected by the effects of the COVID-19 pandemic [11,12,13]. Numerous studies around the world indicate a substantial reduction in student PA, somewhere between 48% and 61%, compared to the years before the advent of COVID-19 [11,14]—with the deficit of physical exercise of moderate and vigorous-intensity being more noticeable in men (−39.2% and −24.6%) than in women (−31.8% and −9.1%) [15].

Reports on students’ quality of life even before the COVID-19 pandemic indicate the presence of other non-communicable diseases, such as mental disorders, depression, anxiety, and stress [16]. Increased physical activity level reduces neuroendocrine stress hormones, cortisol, and epinephrine in women averages 19 years [17]. Such a deficit of stress hormones caused by appropriate PA is a possible factor in improving the unfavorable and disturbed psycho-physical condition [18]. Life circumstances caused by the COVID-19 pandemic have a negative effect on students’ psycho-physical health. The poor psycho-physical condition leads to negative side effects such as increased stress levels, an etiological risk factor for developing anxiety and depressive disorders [19,20,21]. Students can often fail in managing the obligations of their study program, which ultimately leads to psychological problems that represent a genuine risk to their health and aggravate success at the academic level and the development of future careers [22]. Furthermore, the disturbed state of society, i.e., numerous restrictive measures that are in place, causes social disturbance and leads to negative emotional and mental states. Students are a vital part, producing proximate unintentional consequences on the psycho-physical condition of the population’s general health [23,24]. Adapted physical activity, especially during the COVID-19 pandemic, appears to be a universal, readily available drug that alleviates imposed restrictive measures and helps reduce undesirable psycho-physical conditions [12,24,25,26]. Also, studies showed a significant variation in mental health between sexes due to different stress coping mechanisms. It is recognized that women have a higher level of negative emotional states (depression and anxiety) than their male counterparts [12,24]. Moreover, some studies emphasize the difference in physical activity and mental health between students’ education levels. Undergraduate students are more physically active than graduate students [27], which could be associated with their mental health [28,29].

Therefore, this study aimed to specify the prevalence of anxiety, depression, and stress symptoms, as well as physical activity among health-related university students during the second partial COVID-19 lockdown in Croatia, with the objective to explore vulnerabilities of mental well-being that are understudied in our country. Also, it explored the association of gender and study program with depression, anxiety, stress, and physical activity levels. Furthermore, the aim was to study the difference in negative emotional states and physical activity between female and male students, with the objective to corroborate the findings of other studies, as well as to compare and contrast mental health differences between these two groups. This was also in line with the additional aim, where the difference in negative emotional states and physical activity between undergraduate and graduate students has been explored.

## 2. Participants and Methods

### 2.1. Participants

This cross-sectional study, conducted in the period between 26 November and 31 January 2021, included 823 students (153 males and 670 females) from the Faculty of Dental Medicine and Health Osijek of the Josip Juraj Strossmayer University of Osijek, with a response rate of 72.6%. Students were invited to participate in the online research via the official e-mail address, ensuring anonymity and random selection. The student body composition includes female students’ predomination and more students in undergraduate study programs. Participants were divided based on their gender and study program (i.e., undergraduate and graduate). Higher education of health-related students in the Republic of Croatia is conducted through undergraduate and graduate study programs or integrated undergraduate and graduate programs. The undergraduate study program lasts three years, the graduate two years, while the integrated undergraduate and graduate study program lasts six years. Based on such a division of study programs, students are divided into undergraduate (first to the third year of study) and graduate (fourth to the sixth year of study). Nursing and physical therapy students have undergraduate and graduate study programs, while dental medicine students have integrated undergraduate and graduate programs. The Ethical Committee of the Faculty of Dental Medicine and Health Osijek approved the study, and all participants gave written informed consent. The study was conducted online according to the Declaration of Helsinki and its amendments.

### 2.2. Questionnaires

Students were invited to complete a questionnaire containing three parts. In the first part, they were questioned about their socio-demographic (age, gender, height, weight), academic characteristics (study, study program, grade point average (GPA) score), and self-evaluation of mental stress and physical activity. The body mass index (BMI) was calculated as the weight (in kilograms) divided by height in meters squared.

The second part consisted of the Depression Anxiety Stress Scale-21 (DASS-21). The Depression Anxiety Stress Scale-21 questionnaire was used to assess the prevalence of stress, anxiety, and depression symptoms among students. Lovibond and Lovibond created the DASS-21 to measure negative affective conditions and discriminate between anxiety and depression [30]. We used a short, translated to Croatian, and validated version of DASS-21 [31,32]. DASS-21 consists of 21 items that seek to assess three negative emotional states (three subscales), with each state covered with seven items. The items are statements related to the subjective assessment of feelings and behavior over the past week. DASS-21 items are rated on a 4-point Likert-type scale ranging from 0 (does not apply to me at all) to 3 (applies to me very much, or most of the time). The first subscale is depression, which focuses on a bad mood, motivation, and self-esteem. The second subscale is anxiety, based on psychological excitement, panic, and fear. The third subscale is stress, and it focuses on tension and irritability. The result on each subscale is obtained by summing the estimates of the corresponding items and subsequently classified as normal, mild, moderate, severe, and extremely severe [30,33].

Students were asked about their physical activity using the Godin-Shephard Leisure-Time Physical Activity Questionnaire (GSLTPAQ) in the third part. This questionnaire is a short questionnaire with three items frequently used to estimate leisure physical activity. The Godin-Shephard Leisure-Time Physical Activity Questionnaire is a self-administered questionnaire with questions asking for information on how often someone has engaged in mild, moderate, and strenuous physical activity in their spare time (Table 1) [34]. The results obtained from the GSLTPAQ include total weekly physical activity in leisure time, named the leisure score index. It is calculated using corresponding metabolic equivalent according to the formula: (3 × mild) + (5 × moderate) + (9 × strenuous) [35,36]. In this study, a time frame of 7 days (i.e., last week) was used with an activity of at least 15 min. Such instructions were given to examine students’ leisure-time physical activity during the second partial lockdown in Croatia. We used the cut-offs for computing a score related to health as suggested by Godin [35] to divide our sample into two groups (active and insufficiently active). In the analysis of the GSLTPAQ results, only moderate and strenuous components were implemented, so students with a score of 24 or more units were classified as active, while those between 14 and 23 units were classified as insufficiently active. Such a scoring system is applied because it contributes the most to health benefits according to physical activity guidelines for public health [35,36,37]. In this study, the questions of the GSLTPAQ were drawn from a Croatian translation of the GSLTPAQ previously used in similar research [38].

### 2.3. Statistical Analyses

A factor analysis was carried out to estimate the reliability of the instruments, and Cronbach alphas were determined. Numerical variables are presented as mean and standard deviations (SD), and categorical variables are described with absolute and relative frequency. The chi-square and t-test were used to compare the categorical and numerical variables between the gender and study programs. Pearson’s correlation was used to examine the association between depression, anxiety, stress, physical activity, age, GPA, and BMI. In addition, multivariate linear regression analyses were performed to ascertain the independent effects of age, gender, study program, and total GSLTPAQ score on the DASS-21 total score and DASS-21 subscale scores. The *p*-value ≤ 0.05 was considered statistically significant. All analyses were performed using SPSS software (ver. 22.0, SPSS Inc., Chicago, IL, USA).

## 3. Results

### 3.1. Reliability of DASS-21 and GSLTPAQ

Cronbach alphas for the three subscales of the DASS-21 were 0.921, 0.894, and 0.905 for the stress, anxiety, and depression subscale.

The Cronbach alpha in the total sample for the GSLTPAQ was 0.544. Comprehensive data on corrected item-total correlations, factor loadings, and alpha if items deleted of the Croatian GSLTPAQ are shown in Table 1. Table 2 presents inter-item correlations of the Croatian GSLTPAQ items.

### 3.2. Relationship of DASS-21 and GSLTPAQ 

We surveyed a total of 823 students with an average age of 28 ± 9 years. A more significant part of our study participants were female and undergraduate students, in accordance with the student body with the predomination of female students, as well as a higher number of students in undergraduate study programs.

Pearson’s correlations coefficients are presented in Table 3. Depression was significantly positively correlated with anxiety and stress; moreover, anxiety was also positively correlated with stress. However, no significant correlation was observed between negative affective conditions and physical activity.

The results of the multivariate linear regression analyses used to determine the effects of age, gender, study program, and total GALTPAQ score on the DASS-21 are presented in Table 4. Additionally, individual regression analyzes were conducted to ascertain the effects of the mentioned factors on the depression, anxiety, and stress DASS-21 subscales. In terms of physical activity, the only association found was between the female gender and lower GSLTPAQ scores (t = −3.21, 95% CI from −14.31 to −3.45, *p* = 0.001) (Supplementary Table S1).

### 3.3. DASS-21 and GSLTPAQ Related Differences between Gender

Socio-demographic data and self-evaluated mental stress and physical activity are presented in Table 5. During the second partial lockdown, female students reported increased mental stress and decreased physical activity compared to their male counterparts.

Gender-related differences of the DASS-21 and the GSLTPAQ are shown in Table 6. It is observed that female students had significantly higher levels of negative affective conditions than male students. Also, female students scored lower on physical activity than their male counterparts. 

The prevalence of the DASS-21 and GSLTPAQ, divided by categories based on pre-defined cut-points, are shown in Table 7. The majority of the male students were scored within the normal range for symptoms of depression, anxiety, and stress. Two-thirds of female students showed significant anxiety and stress symptoms, while slightly more than half of the female students were depressed. The differences between the two groups are statistically significant. At the same time, two-thirds of female students are insufficiently physically active.

### 3.4. DASS-21 and GSLTPAQ Related Differences between Study Program

Socio-demographic data and self-evaluated mental stress and physical activity are presented in Table 8. During the second partial lockdown, graduate students reported increased mental stress, while there was no significant difference in self-assessment of physical activity during the lockdown.

Study program-related differences of the DASS-21 and the GSLTPAQ are presented in Table 9. It is observed that graduate students had significantly higher levels of stress than undergraduate students. Furthermore, graduate students scored lower on physical activity than undergraduate students.

The prevalence of the DASS-21 and GSLTPAQ, divided by categories based on pre-defined cut-points, are shown in Table 10. The majority of the undergraduate students scored within the normal range for depression, anxiety, and stress symptoms. More than half of graduate students showed significant anxiety symptoms. The differences in depression and stress categories between the two groups are not significant. Further, the majority of undergraduate students were physically active than graduate students.

## 4. Discussion

Data from this study highlight several findings pertinent for the COVID-19 pandemic: (i) the results indicate an insufficient level of physical activity among students, (ii) no significant association was observed between decreased levels of physical activity and the levels of negative emotional states, (iii) prevalence of anxiety, depression, and stress symptoms among health care students are high.

In this study, the psychometric properties of Croatian GSLTPAQ are modest (Cronbach alpha 0.544). However, the results do not differ too much from the scales translated into other languages, where the alpha was about 0.6 [39], which is quite decent considering that the scale is short. A possible reason for the modest Cronbach’s alpha is in observing factor loadings and inter-item correlations. It can be seen that Item 3 correlated the worst with the other items (Table 2) and had the lowest factor loading (0.471, Table 1). The item’s formulation is: ‘How many times on the average do you do mild exercise?’ It is possible that health-related students habitually engage in some mild form of exercise daily, even during clinical practice. These habits make them biased in responding to this item. This might indicate that this questionnaire is not perfect for this population of students, and more research should be done in the broad population. A GSLTPAQ questionnaire was used in Croatia on populations aged 23 to 40 and 53 to 70 years [38]. The results are comparable to the findings from previous studies, and GSLTPAQ was useful for measuring physical activity in leisure time [38]. The DASS-21 is a reliable measure that shows high sensitivity in determining students’ levels of anxiety, depression, and stress symptoms. The internal reliability for the present sample is high. Cronbach alphas for anxiety, depression, and stress subscales were 0.89, 0.90, and 0.92, respectively.

This study was among the first to evaluate the influence of the COVID-19 pandemic on physical activity and mental health among undergraduate and graduate health-related students at the University of Osijek. Students are the most gravely affected population by the current pandemic, which leads to their insecurity, anxiety, and stress [40].

The present study observed that undergraduate students were associated with lower DASS-21 total scores and with anxiety and stress subscales, whereas being younger and female were associated with higher DASS-21 total and subscale scores. Regression analysis showed that being a female and young were independent predictors for more unsatisfactory mental health outcomes in all DASS-21 subscales. Similar to our findings, Elbay et al. showed that young females had a greater risk for developing negative emotional states [41]. Moreover, the female gender was associated with lower physical activity, increasing depressive symptoms [3].

In any case, physical activity in leisure time positively affects mental health and well-being [24]. Reduced physical activity is a risk factor for elevated mental stress [24,25]. That is especially evident during COVID-19 lockdown, as leisure time increases while exercise and sports are limited. Interestingly, there was no correlation between three negative emotional states (depression, anxiety, and stress) and leisure-time physical activity, although that kind of correlation was reported in other studies [24,25].

A possible explanation for the data discrepancy observed in this study is due to much higher levels of depression, anxiety, and stress symptoms (39.5%, 37.1%, 38%) compared to other studies (34.19%, 21.34%, and 28.14%) [42]. At the same time, physical activity levels were similar (40.8% vs. 47.7% and 41.4%) [43,44]. The large difference in negative emotional states may have contributed to the fact that there was no correlation between physical activity and the three negative emotional states in this study. A relatively short questionnaire was used in this study rather than an objective measure of physical activity assessment such as an accelerometer [45]. That could be an additional reason why no correlation was found between physical activity and negative emotional states. In addition to the above, physical activity can be harmful if performed in an inappropriate or very intense manner. Only moderate exercise improves mental health, while intense exercise leads to its worsening, and these variations are associated with depression and anxiety [46]. Also, mental and physical health before the COVID-19 pandemic has a significant direct and indirect impact on physical and mental health during a pandemic [47]. Physical activity explains only 8% of the total effect on mental health [47]. Health-related students were much more physically active, so the current self-assessed reduction in physical activity during the COVID-19 pandemic is not a significant factor in maintaining their physical activity. Research has shown that different types of physical activity do not contribute equally to improving mental health [45]. The effects of the association between physical activity and mental health are small to moderate.

One of the possible factors for a more significant deficit of physical activity is more restrictive measures and complete closure of economic and university activities in the observed area at the survey time [44,48]. At the time of our research, moderate measures were enforced in Croatia. The respondents from our research are exclusively future health professionals who belong to the scientific field of biomedicine and health. During their education, they recognize and adopt the importance of adequate physical exercise and maintain the necessary physical activity levels, with all the direct and indirect benefits of such a lifestyle. It is possible that self-awareness of the importance of movement, especially during the pandemic, has contributed to improved physical activity outcomes in students attending health studies [14,26], such is the case with our research results compared to studies with a broader professional profile of students [43,44]. It is also necessary to take into consideration the different methodologies for assessing the state of physical activity. The GSLTPAQ questionnaire was used in our study, while studies from China used the international physical activity questionnaire [43,44], particularly its short form consisting of seven questions.

Gender has a significant impact on the severity of anxiety, stress, and depression symptoms, as seen in other studies [20,22]. Female students reported higher self-assessment levels of mental stress and reduced physical activity than their male counterparts (even though males had a higher BMI than females in this study). The results also reveal that female students have more significant mental health problems (Table 7). More specifically, women had higher levels of depression, anxiety, and stress based on the DASS-21 scale and were insufficiently active, based on GSLTPAQ, compared to their male counterparts. The possible explanation is that males are doing more physical activity due to a higher BMI than females. Previous studies have shown that female students are more likely to develop depression and anxiety [12,13,16,24].

Furthermore, many studies reported that the female gender is associated with higher negative emotions during the COVID-19 pandemic [12,16,40,49]. Some studies showed the anxiety symptoms prevalence of 57.8% in females [16], similar to our finding of 55.4%. Meanwhile, male students had significantly lower levels of stress symptoms; 68% had normal stress levels than their female counterparts (46%), which is similar to the study of Kecojevic et al. [40]. Women may be more susceptible to environmental stressors than men, and in the circumstances of the COVID-19 pandemic, female students were more likely to express internalized disorders such as stress [40]. Previous studies have identified many stressors contributing to raised stress, anxiety, and depressive symptoms among students [50,51]. Nevertheless, the COVID-19 pandemic is associated with some new and previously seldom-described risk factors. Women usually have higher levels of negative affective conditions such as depression and anxiety than their male counterparts [12,24].

At the same time, graduate students face job insecurity due to layoffs and potential job closures during the pandemic [40,52,53]. Hence, graduate students are expected to have higher levels of negative affective conditions than undergraduate students. The present study found a significant difference in age between undergraduate and graduate students, with graduate students being older than undergraduate students. Graduate students reported higher self-assessment levels of mental stress, while there was no difference in self-assessment levels of physical activity. According to the results of the DASS-21 questionnaire, graduate students had higher levels of all three negative emotional states, but only anxiety levels were significantly different between undergraduate and graduate students. Some other studies have also not found a difference in the prevalence of depression, anxiety, and stress symptoms between undergraduate and graduate students [22,54]. Different studies reported that undergraduate students had a higher prevalence of depression and anxiety symptoms than graduate students [20,55]. These results are in line with Browning et al., whose findings showed that younger students had a greater risk for mental health disorders than older students [49]. A higher proportion of graduate students (63%) were insufficiently active based on GSLTPAQ than undergraduate students (56.1%), which could contribute to the higher prevalence of negative emotional states in graduate students—even though there was no significant difference between their BMI.

Suppose negative emotional states (e.g., depression and anxiety) developed in the early stages of a pandemic remain without intervention (e.g., psychoeducation, psychotherapeutic interventions); in that case, there is a possibility that it could lead to a posttraumatic stress disorder, which can subsequently result in numerous psychosomatic diseases [56]. Compared to our previous research [16], high levels of depressive and anxiety symptoms are similar to those obtained in the pre-pandemic period. During the first lockdown in Croatia, the study conducted on students showed that levels of depression, anxiety, and stress were normal in more than 60% of students during the pandemic. In comparison, higher levels were found in around 20 to 30% of students [55]. A significantly lower prevalence of negative emotional states in the Vulić-Prtorić et al. study [55] could be explained by the fact that the research was conducted at the beginning of the pandemic when we were not fully aware of the pandemic scale and how long we would live under special measure restrictions. In this study, students had a higher prevalence of depression, anxiety, and stress symptoms, possibly because they are health-related students with increased awareness of how the health system operated during the pandemic. Also, most students in this study were women (81.4%), who are known to have higher levels of negative emotional states, contributing to the higher prevalence of depression, anxiety, and stress symptoms. However, many other studies also have a higher number of female participants and got comparable results [12,20,40]. The present study highlights that students are at a high risk for psychological distress during the COVID-19 pandemic; hence, increased and sustained efforts are required to improve their positive mental health and well-being. Universities should offer early detection and tailored prevention programs. Interventions for depression and anxiety among students should be conducted before graduation since it may have long-term effects on their future careers as healthcare professionals.

This study has several limitations. The sample was biased toward women, which is a consequence of the current student body consisting dominantly of female students. Furthermore, the sample was biased towards undergraduate students because they represent the majority of enrolled students. However, our sample is specific and homogeneous because it includes health-related students who have some basic knowledge of virology, the spread of the virus, and how to implement protection measures appropriately. We do not have data on the students’ negative emotional states and physical activity before the COVID-19 pandemic, so we cannot establish whether there is indeed a cause-effect relationship between these observations and the pandemic.

## 5. Conclusions

The study results showed that the prevalence of anxiety, depression, and stress symptoms among health-related university students during the second partial COVID-19 lockdown in Croatia was high. Additionally, the majority of students were insufficiently active. Furthermore, female students had significantly higher levels of negative emotional states than their male counterparts and were significantly less physically active. Graduate students had significantly higher anxiety symptoms and were insufficiently active compared to undergraduate students. It is essential to monitor and promote students’ mental health, particularly in more affected women, to reduce the burden of the pandemic’s adverse effects.

## Figures and Tables

**Table 1:** Item means and standard deviations, corrected item-total correlations, alpha if items deleted and factor loadings of the items of the Croatian GSLTPAQ (N = 823).

Item	Mean ± SD	Corrected Item-Total Correlations	Alpha If Item Deleted	Factor Loadings
During a typical 7-Day period (a week), how many times on the average do you do the following kinds of exercise for more than 15 min during your free time: 1. Strenuous exercise (heart beats rapidly) (e.g., running, jogging, hockey, football, soccer, squash, basketball, cross country skiing, judo, roller skating, vigorous swimming, vigorous long distance bicycling)				
	1.12 ± 1.81	0.483	0.358	0.826
2. Moderate exercise (not exhausting) (e.g., fast walking, baseball, tennis, easy bicycling, volleyball, badminton, easy swimming, alpine skiing, popular and folk dancing)				
	2.45 ± 3.56	0.497	0.217	0.869
3. Mild exercise (minimal effort) (e.g., yoga, archery, fishing from river bank, bowling, horseshoes, golf, snow-mobiling, easy walking)				
	2.99 ± 2.36	0.223	0.621	0.471

**Table 2:** Inter-item correlations of the items of the Croatian GSLTPAQ (N = 823).

Variable	Item 2	Item 3
Item 1	0.557	0.126
Item 2		0.237

**Table 3:** Correlations between age, GPA, BMI, scores of the DASS-21, and Physical Activity (GSLTPAQ) scales (N = 823).

	GPA	BMI	Depression	Anxiety	Stress	Physical Activity
Age	0.037	0.148 **	−0.081 *	−0.041	−0.040	−0.051
GPA		−0.104 *	0.014	0.036	0.039	−0.021
BMI			0.010	0.003	−0.006	−0.039
Depression				0.780 **	0.849 **	0.029
Anxiety					0.830 **	−0.002
Stress						0.012

* *p* < 0.05 Pearson’s r; ** *p* < 0.01 Pearson’s r; GPA—grade point average; BMI—body mass index.

**Table 4:** Multivariate regression analyses on total DASS-21 scores and DASS-21 subscales (N = 823).

	B	SE	t	95% CI	*p*
Total DASS-21					
Age	−0.17	0.06	−2.66	−0.29–(−0.05)	**0.008**
Gender	6.43	1.38	4.66	3.72–9.14	**<0.001**
Study program	2.87	1.18	2.44	0.56–5.18	**0.015**
PA	0.04	0.02	1.71	−0.01–0.08	0.087
Depression					
Age	−0.06	0.02	−2.84	−0.11–(−0.02)	**0.005**
Gender	1.67	0.48	3.44	0.72–2.61	**0.001**
Study program	0.70	0.41	1.69	−0.11–1.51	0.09
PA	0.01	0.01	1.31	−0.01–0.03	0.19
Anxiety					
Age	−0.05	0.02	−2.23	−0.09–(−0.01)	**0.026**
Gender	1.98	0.47	4.24	1.06–2.89	**<0.001**
Study program	0.92	0.39	2.30	0.13–1.70	**0.022**
PA	0.01	0.01	1.71	−0.01–0.03	0.087
Stress					
Age	−0.06	0.02	−2.41	−0.11–(−0.01)	0.016
Gender	2.78	0.52	5.34	1.76–3.81	<0.001
Study program	1.25	0.45	2.82	0.38–2.13	0.005
PA	0.01	0.01	1.78	−0.01–0.03	0.075

B—unstandardized beta coefficient; SE—standard error; CI—confidence interval; PA—physical activity assessed by GSLTPAQ; Gender: 1 = male, 2 = female; Study program: 1 = undergraduate, 2 = graduate. The bold are statistically significant values.

**Table 5:** Socio-demographic data and self-assessment of mental stress and physical activity according to gender.

Variable	Male 153 (18.6%)	Female 670 (81.4%)	All Students 823 (100%)	Test Statistics	df	*p*-Value
Age, years	28 ± 9	28 ± 9	28 ± 9	0.14	821	0.89 *
	Study					
Nursing	102 (66.7%)	548 (81.8%)	650 (79%)	24.09	2	**<0.001 ^†^**
Dental medicine	30 (19.6%)	49 (7.3%)	79 (9.6%)			
Physical therapy	21 (13.7%)	73 (10.9%)	94 (11.4%)			
	Study program					
Undergraduate	95 (62.2%)	358 (57.4%)	453 (55%)	4.26	1	**0.04 ^†^**
Graduate	58 (37.9%)	312 (46.6%)	370 (45%)			
GPA	3.92 ± 0.75	3.97 ± 0.54	3.96 ± 0.58	−0.96	821	0.33 *
BMI	26.52 ± 3.58	23.65 ± 5.59	24.19 ± 5.39	6,07	821	<0.001 *
	Mental stress					
Reduced	8 (5.2%)	25 (3.7%)	33 (4%)	18.16	2	**<0.001 ^†^**
Constant	89 (58.2%)	272 (40.6%)	361 (43.9%)			
Increased	56 (36.6%)	373 (55.7%)	429 (52.1%)			
	Physical activity					
Reduced	84 (54.9%)	382 (57%)	466 (56.6%)	6.79	2	**0.03 ^†^**
Constant	54 (35.3%)	179 (26.7%)	233 (28.3%)			
Increased	15 (9.8%)	109 (16.3%)	124 (15.1%)			

* *t*-test; ^†^ Chi-square test; df—degrees of freedom; GPA—grade point average; BMI—body mass index. The bold are statistically significant values.

**Table 6:** Gender-related differences of students’ scores on the DASS-21 and the GSLTPAQ (N = 823).

Variable	Male (n = 153) Mean ± SD	Female (n = 670) Mean ± SD	All Students (N = 823) Mean ± SD	t	df	*p*-Value *
Depression	4.65 ± 4.91	6.33 ± 5.39	6.02 ± 5.35	−3.54	821	**<0.001**
Anxiety	3.44 ± 4.64	5.58 ± 5.25	5.18 ± 5.20	−4.65	821	**<0.001**
Stress	5.76 ± 5.37	8.64 ± 5.82	8.12 ± 5.84	−5.58	821	**<0.001**
Physical activity	29.33 ± 21.97	20.80 ± 31.43	22.39 ± 30.07	3.18	821	**0.002**

* *t*-test; SD—standard deviation; df—degrees of freedom. The bold are statistically significant values.

**Table 7:** The prevalence of the categories of depressive, anxiety, and stress symptoms according to the DASS-21 and GSLTPAQ. Groups are divided by gender (N = 823).

DASS-21		Males, n (%)	Females, n (%)	All Students, n (%)	t	*p*-Value *
Depression	Normal	92 (60.1)	313 (46.7)	405 (49.2)	−3.01	**0.003**
	Mild	13 (8.5)	80 (11.9)	93 (11.3)		
	Moderate	27 (17.6)	124 (18.5)	151 (18.3)		
	Severe	11 (7.2)	71 (10.6)	82 (10)		
	Extremely severe	10 (6.5)	82 (12.2)	92 (11.2)		
Anxiety	Normal	105 (68.6)	299 (44.6)	404 (49.1)	−5.32	**0.001**
	Mild	16 (10.5)	98 (14.6)	114 (13.9)		
	Moderate	11 (7.2)	62 (9.3)	73 (8.9)		
	Severe	7 (4.6)	55 (8.2)	62 (7.5)		
	Extremely severe	14 (9.2)	156 (23.3)	170 (20.7)		
Stress	Normal	104 (68)	308 (46)	412 (50.1)	−4.87	**<0.001**
	Mild	15 (9.8)	83 (12.4)	98 (11.9)		
	Moderate	10 (6.5)	93 (13.9)	103 (12.5)		
	Severe	19 (12.4)	101 (15.1)	120 (14.6)		
	Extremely severe	5 (3.3)	85 (12.7)	90 (10.9)		
PA **	Active	82 (53.6)	254 (37.9)	336 (40.8)	4.47	**<0.001**
	Insufficiently active	71 (46.4)	416 (62.1)	487 (59.2)		

* *t*-test with 821 degrees of freedom; ** PA—physical activity assessed by the GSLTPAQ. The bold are statistically significant values.

**Table 8:** Socio-demographic data and self-assessment of mental stress and physical activity according to the study program.

Variable	Undergraduate 453 (55%)	Graduate 370 (45%)	Test Statistics	df	*p*-Value
Age, Years	25 ± 8	32 ± 9	−13.29	816	<0.001 *
	Study				
Nursing	357 (78.8%)	293 (79.5%)	0.35	2	0.84 ^†^
Dental medicine	47 (9.9%)	32 (8.8%)			
Physical therapy	51 (11.3%)	43 (11.8%)			
	Gender				
Male	95 (21%)	58 (15.3%)	4.26	1	**0.04 ^†^**
Female	358 (79%)	312 (84.7%)			
GPA	3.88 ± 0.62	4.06 ± 0.52	−4.55	816	<0.001 *
BMI	23.95 ± 6.18	24.47 ± 4.24	−1.37	816	0.17 *
	Mental stress				
Reduced	23 (5.1%)	10 (2.5%)	14.27	2	**0.01 ^†^**
Constant	219 (48.3%)	142 (38.4%)			
Increased	211 (46.6%)	218 (59.2%)			
	Physical activity				
Reduced	242 (53.4%)	224 (60.8%)	4.55	2	0.10 ^†^
Constant	138 (30.5%)	95 (25.2%)			
Increased	73 (16.1%)	51 (14%)			

* *t*-test; ^†^ Chi-square test; df—degrees of freedom; GPA—grade point average; BMI—body mass index. The bold are statistically significant values.

**Table 9:** Study program-related differences of students’ scores on the DASS-21 and the GSLTPAQ (N = 823).

Variable	Undergraduate (n = 453) Mean ± SD	Graduate (n = 370) Mean ± SD	t	df	*p*-Value *
Depression	5.91 ± 5.45	6.19 ± 5.24	−0.75	816	0.45
Anxiety	4.89 ± 5.11	5.57 ± 5.31	−1.86	816	0.06
Stress	7.69 ± 5.91	8.64 ± 5.75	−2.35	816	**0.02**
Physical activity	24.06 ± 36.08	20.30 ± 20.35	1.77	816	**0.04**

* *t*-test; SD—standard deviation; df—degrees of freedom. The bold are statistically significant values.

**Table 10:** The prevalence of the categories of depressive, anxiety, and stress symptoms according to the DASS-21 and GSLTPAQ. Groups are divided by study program (N = 823).

DASS-21		Undergraduate, n (%)	Graduate, n (%)	t	*p*-Value *
Depression	Normal	236 (52.1)	169 (45.7)	−0.77	0.35
	Mild	39 (8.6)	54 (14.6)		
	Moderate	81 (17.9)	70 (18.9)		
	Severe	47 (10.4)	35 (9.5)		
	Extremely severe	50 (11)	42 (11.3)		
Anxiety	Normal	231 (51)	173 (46.8)	−1.72	**0.01**
	Mild	59 (13)	55 (14.9)		
	Moderate	45 (9.9)	28 (7.6)		
	Severe	39 (8.6)	23 (6.2)		
	Extremely severe	79 (17.4)	91 (24.5)		
Stress	Normal	239 (52.8)	173 (46.8)	−1.76	0.08
	Mild	49 (10.8)	49 (13.2)		
	Moderate	57 (12.6)	46 (12.4)		
	Severe	65 (14.3)	55 (14.9)		
	Extremely severe	43 (9.5)	47 (12.7)		
PA **	Active	199 (43.9)	137 (37)	1.97	**0.04**
	Insufficiently active	254 (56.1)	230 (63)		

* *t*-test with 816 degrees of freedom; ** PA—physical activity assessed by the GSLTPAQ. The bold are statistically significant values.

## Data Availability

The dataset generated during the study is available from the corresponding author on reasonable request.

## Supplementary Materials

The following are available online at https://www.mdpi.com/article/10.3390/healthcare9070801/s1, Table S1: Multivariate regression analyses on total GSLTPAQ scores (N = 823).
