# Supplementary material for: Mental Health and Physical Activity in Health-Related University Students during the COVID-19 Pandemic
Source: Healthcare (Basel). 2021 Jun 25;9(7):801. doi: 10.3390/healthcare9070801 (PMC8304952; doi:10.3390/healthcare9070801)
Supplement: Supplementary file 1 [file healthcare-09-00801-s001.zip › healthcare-1258257-supplementary.pdf]

Table S1. Multivariate regression analyses on total GSLTPAQ scores (n=823).

	B	SE	t	95% CI	p
**Total GSLTPAQ**					
Age	-0.16	0.12	-1.37	-0.38-0.07	0.17
Gender	-8.83	2.74	-3.22	-14.20-(-3.45)	**0.001**
Study program	-2.57	2.35	-1.09	-7.17-2.04	0.274
Total DASS-21	0.04	0.02	1.71	-0.01-0.08	0.087
Depression	0.31	0.37	0.79	-0.45-1.07	0.426
Anxiety	-0.30	0.37	-0.81	-1.04-0.43	0.417
Stress	0.17	0.40	0.42	-0.62-0.95	0.673

B – unstandardized beta coefficient; SE – standard error; CI – confidence interval; Gender: 1=male, 2=female; Study program: 1=undergraduate, 2=graduate. The bold is statistically significant value.
